# Nutcracker syndrome mimicking new daily persistent headache: A case report

**DOI:** 10.1177/0333102420918554

**Published:** 2020-04-15

**Authors:** Anker Stubberud, Sanjay Cheema, Erling Tronvik, Manjit Matharu

**Affiliations:** 1 Headache and Facial Pain Group, UCL Queen Square Institute of Neurology and National Hospital for Neurology and Neurosurgery, London, United Kingdom; 2 Department of Neuromedicine and Movement Sciences, NTNU Norwegian University of Science and Technology, Trondheim, Norway; 3 Department of Neurology, St. Olavs Hospital, Trondheim, Norway

**Keywords:** Superior mesenteric artery syndrome, Nutcracker syndrome, surgery, CSF pressure

## Abstract

**Introduction:**

Compression of the duodenum and left renal vein between the aorta and superior mesenteric artery usually leads to symptoms of proximal bowel obstruction or hematuria and, more rarely, nonspecific mild headaches.

**Case:**

A young woman presented with new daily persistent headache refractory to numerous pharmacological treatments, onabotulinumtoxinA, nerve blocks, and occipital nerve stimulation. Following several years of daily severe headache, worsening abdominal pain and intolerance for food intake led to the discovery of aortomesenteric compression. Surgical treatment gave prompt improvement in gastric symptoms but also essentially resolved the headache.

**Conclusion:**

This is the first description of new daily persistent headache in association with aortomesenteric compression as well as marked improvement of headache following aortomesenteric decompression. In patients with new daily persistent headache and orthostatic symptoms one may consider a differential diagnosis of Nutcracker syndrome, especially in patients with comorbid hypermobility syndromes, hematuria or gastric symptoms.

## Introduction

Compression of the duodenum and left renal vein between the aorta and superior mesenteric artery (SMA) leads to SMA syndrome and Nutcracker syndrome, respectively. SMA syndrome usually presents with symptoms of proximal small bowel obstruction (1). Nutcracker syndrome usually presents with hematuria, flank pain, varicocele, orthostatic proteinuria, or orthostatic intolerance. Headaches are known to occur with Nutcracker syndrome (2), but neither syndrome has an established association with new daily persistent headache (NDPH). Here we describe a case of long-standing medically refractory NDPH that completely resolved upon aortomesenteric decompression.

## Case

A 17-year-old woman was first seen in the tertiary Headache Centre at the National Hospital for Neurology and Neurosurgery, UK, in August 2011 with complaints of a persistent headache from 14 January 2011 onwards. There were no known precipitants at the onset of the headache. The headache developed gradually during the day. She described a severe continuous bilateral headache localised over the forehead, temple and vertex with pressing and sharp qualities. The headache was associated with photophobia and motion sensitivity, but no other migrainous symptoms. There were no associated cranial autonomic features. She had one episode of 5 days with numbness, tingling and pain in her hands and feet, but had no other symptoms suggestive of aura. The headache had no relation to posture, and there were no features suggestive of cerebrospinal fluid pressure dysregulation, temporomandibular dysfunction or cervicogenic headache. Prior to onset of the daily headache, she described long-standing mild and featureless headaches that lasted 2–3 hours and occurred 5–10 times per year. Neurological examination was unremarkable and there was no evidence of papilloedema.

She had developed anxiety and depression secondary to the highly disabling headache. She had recently been diagnosed with hypermobile Ehlers Danlos Syndrome (hEDS). She had experienced orthostatic disturbances since age 12 and was diagnosed with postural orthostatic tachycardia syndrome (POTS). Her POTS was well controlled on fludrocortisone, midodrine and octreotide. She was also on olanzapine for her mood disorder. The patient had a family history of migraine and hypermobility.

Prior to referral, MRI scans of the head (with gadolinium contrast) and cervical spine as well as magnetic resonance angiography were normal. Previous drug trials initiated by other health professionals including citalopram, amitriptyline, sertraline, duloxetine, venlafaxine, metoprolol and pizotifen were ineffective for the headaches. The patient tried numerous treatments over the next few years including sodium valproate, topiramate, pregabalin, gabapentin, methysergide, memantine, intravenous dihydroergotamine and onabotulinumtoxinA, which were all ineffective. Multiple cranial nerve blocks including the supraorbital, supratrochlear, proximal and distal auriculotemporal, as well as greater and lesser occipital nerves did not provide any relief. A wide range of triptans and over-the-counter painkillers were also ineffective. The headaches remained unchanged, present every day, with 20 days of moderate intensity per month and 10 days of severe intensity per month, and a substantially disruptive influence on her life. She had an occipital nerve stimulator (ONS) implanted in September 2016 which worsened the headaches. Fludrocortisone, midodrine and octreotide were discontinued prior to the ONS implant with no worsening in POTS symptoms.

From March 2017 the patient developed gradually worsening gastric symptoms. She then weighed 66 kilograms and her height was 164 centimeters. It was at this point noted that she had been troubled with diarrhea for the previous 2 years. In June 2017, she was admitted to her local hospital due to dehydration and malnutrition caused by severe abdominal pain and intolerance for food intake. A few mouthfuls of food and even intake of water could provoke abdominal pain lasting for hours. The gastric symptoms were initially interpreted as EDS-related dysmotility. The symptoms worsened and by August 2017 she had lost 18 kg of weight, required parenteral nutrition and was wheelchair-bound due to severe cachexia. Abdominal ultrasonography was performed by the gastroenterologists in August 2017 and revealed severe Nutcracker syndrome of the left renal vein, May-Thurner constellation (iliac vein compression), compression of the coeliac trunk, and SMA syndrome. Renal vein velocity was 208 cm/s (grossly elevated) and there was marked hypoperfusion of the left renal cortex. An abdominal CT scan and an angiogram of the mesenteric vessels confirmed the diagnoses, explaining the patient’s gastric symptoms. No direct abdominal venogram was completed. On 28 November 2017, a left renal vein transposition and gastrojejunostomy was performed, giving prompt improvement in her gastric symptoms. More surprisingly, the patient experienced dramatic improvement in her headaches and was for the first time in 7 years essentially headache-free.

Following the operation, the patient reported an immediate and lasting resolution of the headache characterising her NDPH. However, she did have some residual occipital pain related to the ONS and reported an episodic bi-frontal, moderate intensity headache often related to her menstrual cycle and travel. The latter was interpreted as a probable episodic migraine transformed from the NDPH, occurring two to six times per month. She also reported worsening of her POTS symptoms after the vascular decompression. Fludrocortisone and midodrine were reinitiated prior to the vascular decompression surgery, and ivabradine was initiated 2 months later. Ivabradine was beneficial for the POTS symptoms and later allowed tapering of fludrocortisone and midodrine.

In August 2018 the ONS was explanted, leading to resolution of the occipital pain. However, 1 month after the explant the patient reported an orthostatic headache. She described moderate intensity bi-occipital headache within 15 minutes of being upright and resolution within 10 minutes of lying down. Bending over occasionally led to a clear nasal discharge. Upon careful questioning it did however appear that both the nasal discharge and occipital orthostatic-type headache had been present since February 2018, possibly earlier. MRI scan of the head and whole spine (with contrast), magnetic resonance angiography (intracranial and extracranial) and magnetic resonance venography were normal. There were no radiological features indicative of a low CSF volume state and no intracranial vascular abnormalities. Analysis of the nasal discharge was negative for CSF proteins. No formal CSF pressure measurement was made. The orthostatic headache resolved spontaneously by December 2018. Since the start of 2019, the patient has had episodic migraine without aura on 5–6 days per month, which she is able to treat readily with triptans and high dose aspirin.

The patient has consented to patient data being published in this case report.

## Discussion

SMA syndrome is most commonly caused by extreme weight loss or corrective spinal surgery for scoliosis (3). The Nutcracker syndrome shares the same etiologies, but is also seen with renal ptosis, vascular abnormalities and intraabdominal tumours. However, the coexistence of both syndromes is very rare (4), and to our knowledge there are no cases describing improvement of NDPH upon surgical correction of these syndromes.

Headache has been described as a symptom accompanying Nutcracker syndrome, mostly in children and adolescents (2,5,6). In one of the studies, 14 of 16 children had headache, but all had resolution of their headaches upon a trial of low dose acetylsalicylic acid (2). The authors propose that antiplatelet therapy lowers blood viscosity, which allows increased perfusion through the stenotic left renal vein, thereby decreasing prestenotic pressure, collateral pressure and shear stress. This leads to a reversal of vessel wall inflammation, edema and thromboses, which results in a drop in venous pressure around the spine and dural sac and thereby lowers intracranial pressure (2). This notion of headache pathophysiology is supported by the fact that induced venous congestion, such as the Queckenstedt manoeuvre and Valsalva test, aggravates headaches, most likely through peripheral nociception in veins (7). In the current case, the left renal vein transposition could have had a similar effect as antiplatelet therapy on venous and spinal fluid pressure and thereby explain the transient orthostatic type headache the patient experienced, which could have been due to rebound intracranial hypotension, though the differential diagnosis included headache due to POTS. Furthermore, it is possible that vasoconstrictors such as triptans and methysergide should have been avoided as it could have worsened the already hypo-perfused renal cortex.

Headaches seen with Nutcracker syndrome are usually part of a constellation of symptoms including abdominal pain, tachycardia and orthostatic intolerance. Orthostatic intolerance is usually a prominent symptom and has in fact been suggested as one of three clinical subtypes of Nutcracker syndrome (8). The onset of orthostatic intolerance may precede the diagnosis by many years (6), which indeed was the case with this patient. The patient had experienced orthostatic symptoms since the age of 12, indicating that compression of the left renal vein may have been present long before the headache and the gastric symptoms. On the other hand, SMA syndrome often presents with intermittent symptoms. The patient had intermittent diarrhea for 2 years and pain provoked by food intake, but no dietary-related fluctuations of headache. This could be explained by the headache being mainly associated with vascular compression, and not the duodenal compression.

In addition, several other factors may have contributed to this patient’s persistent headache. Firstly, it is possible that triggers such as infections, extracranial surgery and stressful life events can incite an underlying disposition for NDPH, albeit that the patient did not identify any such factors (9). Secondly, it is also suggested that hypermobility and EDS is a predisposing factor for NDPH (10), while the underlying mechanisms are largely unknown. Thirdly, POTS and NDPH are also known to co-occur (11), but also here the body of research is too limited for definite conclusions. The patient had no known triggering factors but suffered from both EDS and POTS, possibly predisposing for NDPH. Finally, one study indicates that hypermobility poses an increased risk for vascular compression syndromes (12), which indicates an etiological differential of Nutcracker syndrome should be considered in similar cases.

In conclusion, it is tempting to offer the following explanation: From young age, a progressing left renal vein compression gave orthostatic disturbances, which later was diagnosed as POTS. At some point, the increasing renal vein compression, venous congestion and increased dural sac pressure reached a threshold resulting in NDPH in a predisposed individual. The aortomesenteric compression was only made evident after the debut of gastric symptoms. Surgical correction reversed the venous congestion and the persistent headache, followed by a transient relative low spinal fluid pressure giving an orthostatic-type headache. Eventually, spinal fluid pressure stabilised, and the patient was left with a probable episodic migraine that might have been present all along. Even though the causality is hard to determine based on a single case, the constellation of headache, orthostatic symptoms, gastroenterological symptoms and hypermobility should point towards a diagnosis of abdominal vascular compression and prompt Doppler ultrasonography of renal vein flow velocity or a contrast abdominal CT scan.

## Clinical implications

- This is the first literature report of co-existing superior mesenteric artery syndrome and Nutcracker syndrome where surgical correction led to a prompt relief of refractory new daily persistent headache.
- In patients with refractory new daily persistent headache and orthostatic symptoms, one may consider a differential diagnosis of Nutcracker syndrome, especially in children, patients with comorbid hypermobility syndromes, and in the presence of hematuria or gastric symptoms.
- In patients with confirmed Nutcracker syndrome and headache, a trial of low-dose acetylsalicylic acid may be attempted.
